# Genomic comparative analysis of *Enterobacter asburiae* harbouring a conjugative *bla*_IMI−6_-plasmid isolated from a public garden in Switzerland

**DOI:** 10.1007/s10096-025-05131-7

**Published:** 2025-04-23

**Authors:** Yvonne Spahr, Andrea Endimiani, Vincent Perreten

**Affiliations:** 1 Division of Molecular Bacterial Epidemiology & Infectious Diseases, Institute of Veterinary Bacteriology, Vetsuisse Faculty, University of Bern, Bern, Switzerland; 2 Institute for Infectious Diseases (IFIK), University of Bern, Bern, Switzerland; 3 Division of Molecular Bacterial Epidemiology & Infectious Diseases, Institute of Veterinary Bacteriology, University of Bern, Länggassstrasse 122, Bern, CH-3012 Switzerland

**Keywords:** Carbapenemase, *bla*_IMI−6_, *E. asburiae*, Antibiotic resistance, Environment, IncFII(Yp)

## Abstract

Ambler class A IMI enzymes are minor carbapenemases primarily associated with the *Enterobacter cloacae* complex. Screening soil surfaces of a well-visited public garden revealed an IMI-6-producing *Enterobacter asburiae* ST657 near a trash can. The strain harboured a 163-kb conjugative IncFII(Yp) plasmid containing *bla*_IMI−6_ and putative virulence genes. WGS comparative analysis with other clinical and non-clinical *E. asburiae* ST657 showed that it was genetically related to strains from a patient in France (62 ΔSNPs) and from retail salad in Switzerland (79 ΔSNPs). This finding suggests possible trans-sectoral dissemination of IMI-producing bacteria raising concerns since they could further spread into the community.

**Supplementary Information:**

The online version contains supplementary material available at 10.1007/s10096-025-05131-7.

IMI-like enzymes are a minor group of carbapenemases that belong to the Ambler Class A, as they are less frequently detected than the widely spread carbapenemases (e.g., IMP, KPC, NDM, OXA-48 variants and VIM) [1]. They confer resistance to penicillins, early generation cephalosporins and carbapenems, sparing broad-spectrum cephalosporins and are usually associated with the *Enterobacter cloacae* complex (ECC) [1]. The environment is presumed to be the reservoir of species producing these enzymes, in particular the aquatic ecosystems and their presence is suspected to be underestimated in clinical settings [1]. IMI carbapenemases have been located on both chromosome and plasmids [1].

In August 2024, an IMI-6-producing *Enterobacter asburiae* (strain 19YS-C) was isolated from the soil surface around a trash bin in a public garden in Switzerland. The park is visited by various social groups like families, school or kindergarten groups, joggers, homeless people and dogs with their owners as well as wild animals as it borders the city, a forest and a university referral companion animal hospital. The companion animal hospital is about 60 m from the park, separated by a road. In particular, this veterinary institution has been facing hospital-acquired infections with different OXA-48-like carbapenemase-producing Enterobacterales (CPE) since 2018, and in some cases, animals remained temporarily gut colonized after discharge [2–4]. Notably, before returning home, some hospitalized dogs are occasionally taken for a walk in the public garden opposite the hospital, which prompted us to screen this public garden for the presence of CPE.

The garden soil surface of 12 different areas of 6 m^2^ each were sampled with a sterile wipe, which was applicated in multiple locations over to the ground surface. The wipes were then introduced into a bottle containing 200 ml Mueller-Hinton (MH) broth and incubated at 37 °C for 18 h without shaking. One loopful (10 µl) of the liquid was spread on both CHROMID CARBA and OXA-48 (bioMérieux) selective agar plates and incubated at 37 °C overnight. As a result, suspected CPE were only obtained on plates of the area around a trash bin. The colonies exhibited the same coloration and morphology, and 10 were submitted to Matrix-Assisted Laser Desorption/Ionization Time-of-Flight Mass Spectrometry (MALDI-TOF MS; Bruker) identification to avoid missing other CPE. All were identified as *Enterobacter cloacae* complex and one of them was streak out on trypticase soy agar containing 5% sheep blood (Becton-Dickinson) and carbapenemase production was assessed with the home-made Blue-Carba test [5], an alternative to the commercially available RAPIDEC^®^ CARBA NP (bioMérieux) test [6]. Antimicrobial susceptibility testing (AST) was performed in MH broth using the microdilution Sensititre™ EUVSEC2 and EUVSEC3 plates (Thermo Fisher), except for fosfomycin which was tested using ETEST^®^ (bioMérieux). The isolate named 19YS-C was non-susceptible to ampicillin, cefoxitin, ertapenem, imipenem, meropenem and exhibited high MIC to fosfomycin (Table 1).

**Table 1:** Minimum inhibitory concentrations (MIC) of *E. asburiae* 19YS-C, *E. coli* J53dR1, *E. coli* transconjugant TC19YS containing the *bla*_IMI−6_ harbouring plasmid p19YS-IMI-6 from *E. asburiae* 19YS-C

Antimicrobials	MIC (mg/l)		
	*E. asburiae* 19YS-C (donor)	*E. coli* J53dR1 (recipient)	*E. coli* TC19YS (*E. coli* J53dR1 with p19YS-IMI-6)
Cefoxitin^b^	> 64 (R)	4 (S)	8 (S) (*bla*_IMI−6_)
Ertapenem^a^	> 2 (R)	≤ 0.015 (S)	0.5 (S) (*bla*_IMI−6_)
Imipenem^a^	> 16 (R)	0.25 (S)	8 (R) (*bla*_IMI−6_)
Meropenem^a^	16 (R)	≤ 0.03 (S)	1 (S)^2^ (*bla*_IMI−6_)
Ceftazidime^a^	≤ 0.25 (S)	≤ 0.25 (S)	0.5 (S) (*bla*_IMI−6_)
Cefepime^a^	≤ 0.06 (S)	≤ 0.06 (S)	≤ 0.06 (S)
Cefotaxime + clavulanate	0.5/4 (NA)	≤ 0.06/4 (NA)	≤ 0.06/4 (NA)
Ceftazidime + clavulanate	0.25/4 (NA)	≤ 0.12/4 (NA)	≤ 0.12/4 (NA)
Cefotaxime^a^	≤ 0.25 (S)	≤ 0.25 (S)	≤ 0.25 (S)
Temocillin^a^	4 (NA)	8 (NA)	8 (NA)
Ampicillin^a^	> 32 (R)	4 (S)	> 32 (R) (*bla*_IMI−6_)
Ciprofloxacin^a^	0.03 (S)	≤ 0.015 (S)	≤ 0.015 (S)
Azithromycin	8 (NA)	4 (NA)	4 (NA)
Amikacin^b^	≤ 4 (S)	≤ 4 (S)	≤ 4 (S)
Gentamicin^b^	≤ 0.5 (S)	≤ 0.5 (S)	≤ 0.5 (S)
Tigecycline^a^	0.5 (NA)	≤ 0.25 (NA)	≤ 0.25 (NA)
Chloramphenicol^b^	≤ 8 (S)	≤ 8 (S)	≤ 8 (S)
Colistin^a^	2 (S)	≤ 1 (S)	≤ 1 (S)
Nalidixic acid^b^	≤ 4 (NA)	≤ 4 (NA)	≤ 4 (NA)
Tetracycline^b^	≤ 2 (S)	≤ 2 (S)	≤ 2 (S)
Trimethoprim^b^	0.5 (NA)	≤ 0.25 (NA)	≤ 0.25 (NA)
Sulfomethoxazole^b^	≤ 8 (NA)	≤ 8 (NA)	<=8 (NA)
Fosfomycin^b^	> 512 (NA)	0.25 (NA)	0.25 (NA)

Abbreviations: R, resistant; S, susceptible; NA, not applicable

^1^The MICs were interpreted using the EUCAST^a^ [18] and CLSI recommendations^b^ [19]

^2^above the screening value of 0.125 mg/l

Whole-genome sequencing (WGS) of 19YS-C was performed using both Illumina and Oxford Nanopore Technologies (ONT) as previously described [7]. A hybrid assembly was generated using Unicycler v0.5.0 (https://github.com/rrwick/Unicycler) with Illumina short and ONT long reads, followed by a final polishing step with Illumina reads (Polypolish v0.6.0, https://github.com/rrwick/Polypolish). The assembly was quality controlled with Quast v5.2.0 (https://github.com/ablab/quast) and annotated with the NCBI Prokaryotic Genome Annotation Pipeline (PGAP) v.6.9 (https://github.com/ncbi/pgap). Sequencing statistics and genomic characteristics are available in Table S1 (Supplementary Information). The complete genome and both short and long reads were deposited into GenBank under BioProject PRJNA1214994 with SRA acc. no. SRR32105527 and SRR32105526 and GenBank acc. no. CP179916 and CP179917.

Sequence type (ST) was determined using PubMLST (https://pubmlst.org/). Screening for antimicrobial resistance genes (ARGs) and identification of plasmid incompatibility (Inc) groups were carried out using ABRicate v1.0.1 (https://github.com/tseemann/abricate) against the databases ResFinder and PlasmidFinder, respectively. The NCBI tool BLASTn (https://blast.ncbi.nlm.nih.gov/Blast.cgi) was used to search for similar plasmids deposited in GenBank, which were subsequently used for comparative analysis using clinker v0.0.28 (https://github.com/gamcil/clinker). Strain 19YS-C was identified as *E. asburiae* using the Type (Strain) Genome Server (TYGS) (https://tygs.dsmz.de/) with a dDDH d4 of 75% and by OrthoANIu (https://www.ezbiocloud.net/tools/ani) which calculated an average nucleotide identity (ANI) of 97.06% with *E. asburiae* type strain ATCC 35,953 (GenBank acc. no. GCA_001521715.1). The shared identity between the 16 S rRNA of 19YS-C and ATCC 35,953 was 99.55% based on MAFFT pairwise alignment (Geneious Prime 2025.0.3).

*E. asburiae* 19YS-C belonged to ST657 and consisted of a 4,730,936 bp chromosome and one 162,948 bp plasmid (p19YS-IMI-6). ARGs explaining the fosfomycin and β-lactam resistance profile were located on the chromosome (*bla*_ACT−10_, *fosA*) and on p19YS-IMI-6 (*bla*_IMI−6_). Plasmid p19YS-IMI-6 belonged to IncFII(Yp) plasmids and carried virulence genes for a type VI secretion system (T6SS) and type 1 pilus formation. Five plasmids from GenBank [i.e., pSTS63C-A (CP162151), pIMI-6 (KX786187), pRJ46C (KT225520), pGA45 (KT780723), and pHURS_212964 (JAPHQK010000008.1)] shared a conserved backbone region with p19YS-IMI-6, containing a conjugal transfer region and genes for T6SS. All plasmids carried *bla*_IMI−6_, a copper resistance gene and two regions for type 1 pilus formation in the accessory region, except pGA45 which lacked the type 1 pilus genes. These plasmids were detected in clinical and environmental samples including food from different bacterial species, in different countries and years (Fig. 1) [8–10].

**Fig. 1:**
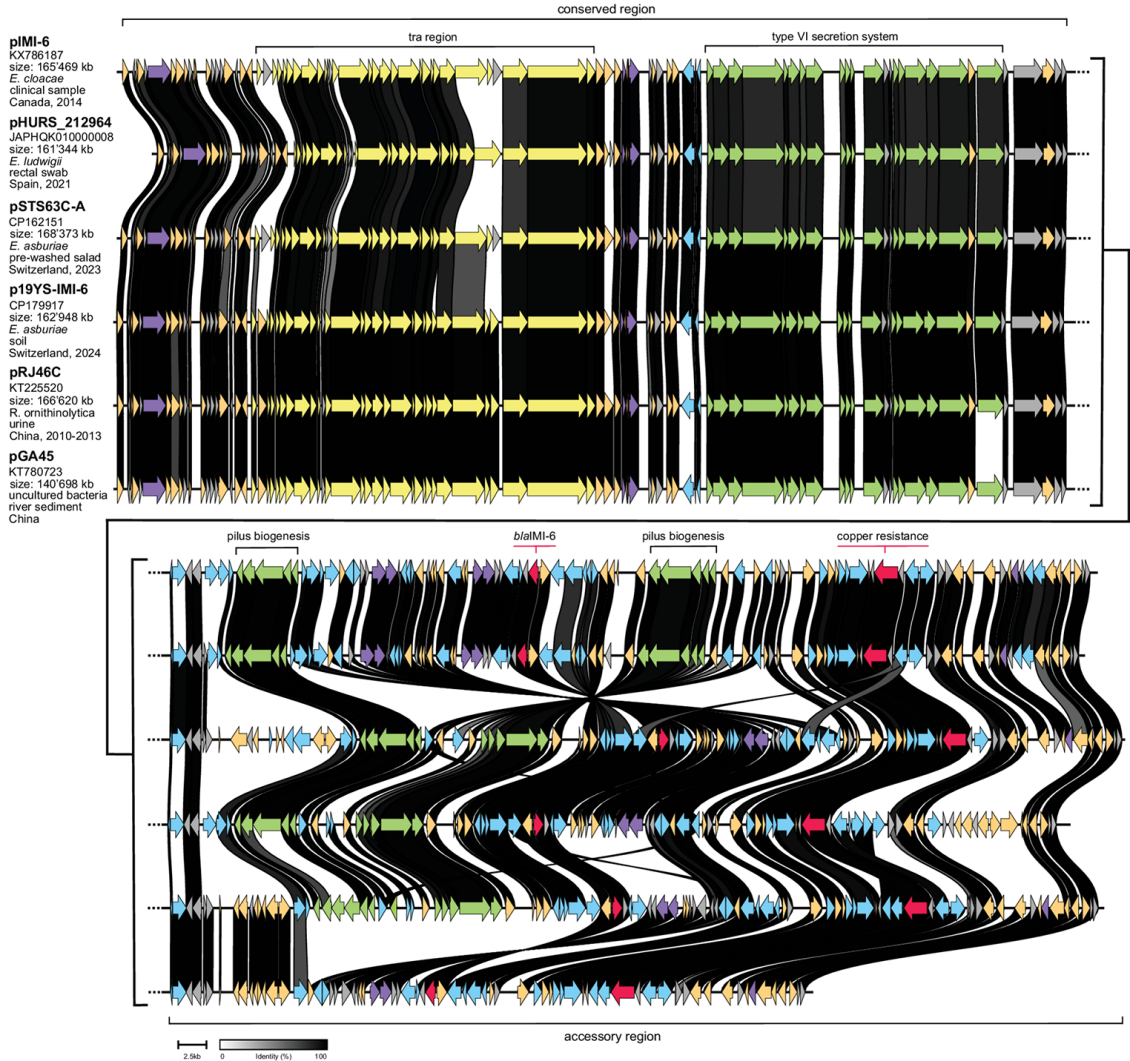
Comparison of plasmid p19YS-IMI-6 to five plasmids harbouring *bla*_IMI−6_ of different bacterial origin. The five plasmids were obtained from GenBank: pSTS63C-A, *Enterobacter asburiae*, pre-washed salad, Switzerland, 2023, 91% coverage, 99.83% identity (GenBank acc. no. CP162151); pIMI-6, *Enterobacter cloacae*, clinical sample, Canada, 2014, 93% coverage, 99.93% identity (KX786187); pRJ46C, *Raoultella ornithinolytica*, clinical sample, China, between 2010 to 2013, 90% coverage, 99.88% identity (KT225520); pGA45, unspecified bacteria, river sediment, China, 76% coverage, 99.79% identity (KT780723); pHURS_212964, *E. ludwigii*, clinical sample, Spain, 2021, 93% coverage, 93.87% identity (JAPHQK010000008.1, draft genome). Plasmid name, GenBank accession numbers, size as well as species, source, country and year of isolation are given in the illustration. For the genes there is a colour code as follows: genes associated with resistance in red, genes associated with virulence in green, genes associated with conjugative gene transfer in yellow, IS elements and transposases in blue, other genes in orange and hypothetical genes and genes of unknown function in grey. All plasmids were newly annotated with PGAP v.6.9 and the figure was generated with clinker v0.0.28, with the identity threshold set to 0.6 between the genes. Adobe Illustrator 2024 was used to adapt the figure

Transferability of p19YS-IMI-6 was tested by filter mating using the sodium azide- and rifampicin-resistant *E. coli* strain J53dR1 as recipient as previously described [11]. Transconjugants were selected on MH agar plates containing 100 µg/ml sodium azide and 50 µg/ml ampicillin. Counter-selection on MH agar plates supplemented with 50 µg/ml rifampicin and 50 µg/ml ampicillin was performed to exclude spontaneous mutants of the donor strain. Plasmid p19YS-IMI-6 was transferable into *E. coli* with a frequency of 3 × 10^− 7^ transconjugants per recipient and the transconjugants were verified by species identification using MALDI-TOF MS and PCR detection of the *bla*_IMI−6_ gene [12].

Single nucleotide polymorphisms (SNP) were called using Snippy v4.6.0 (https://github.com/tseemann/snippy) considering the 19 genomes of *E. asburiae* ST657 available from GenBank (accessed on March 25th, 2025), one strain available from the European Nucleotide Archive (ENA) (accessed March 25th, 2025), and using 19YS-C as reference. The recombination events were filtered with Gubbins v3.3.1 (https://github.com/nickjcroucher/gubbins) and IQ-TREE v2.3.6 (https://github.com/iqtree/iqtree2) was used to reconstruct a cgSNP-based phylogenetic tree. Of the 20 *E. asburiae* ST657 sequences deposited in the databases, 16 were from strains isolated from humans, while 2 were from environmental sources and 2 from vegetables (Fig. 2). *E. asburiae* 19YS-C appeared to be closely related to strain 229I2 isolated from a rectal swab of a human patient in France in 2019 (62 ΔSNPs) [13] and to strain STS63-C detected in a prewashed salad in Switzerland in 2023 (70 ΔSNPs) [8]. These three strains were distantly related (> 206 ΔSNPs) to other strains from France, Sweden, Germany, Japan, China, Taiwan and the USA (Fig. 2). The three closely related strains 19YS-C, STS63-C and 229I2 all harbored *bla*_IMI−6_. Six other strains also contained a carbapenemase, namely IMI-6 in two strains from France, IMI-2 in strains from France and Sweden, IMI-19 in one strain from France, and FRI-4 in one strain from Japan (Fig. 2).

**Fig. 2:**
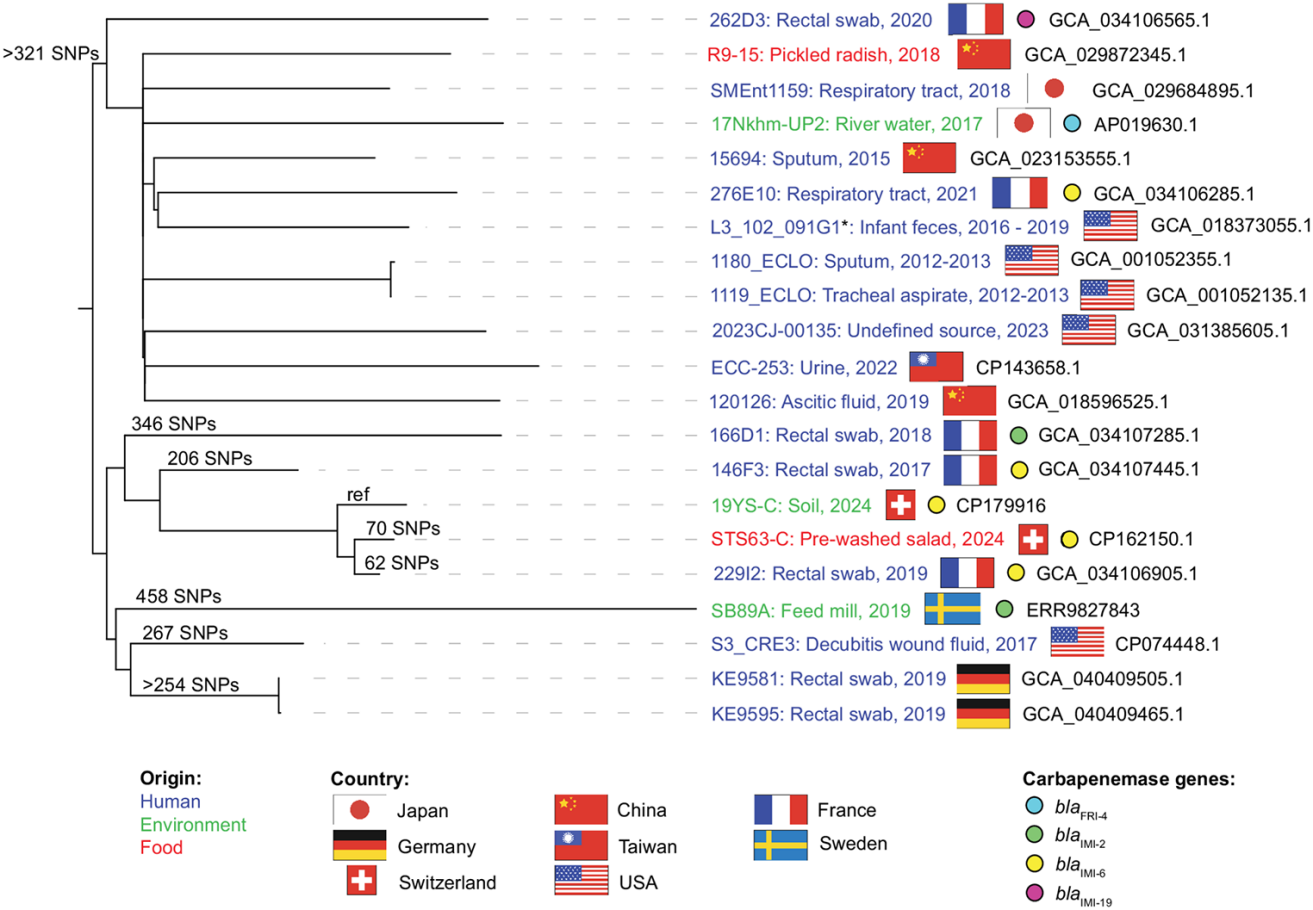
CgSNP-based phylogenetic tree of *Enterobacter asburiae* ST657. Twenty publicly available genomes from GenBank were used for cgSNP analysis against the reference (ref) 19YS-C. The SNPs are always in relation to the reference. The name, GenBank accession number and host of the strain as well as source, country and year of isolation, if available, are presented in the figure. If a carbapenemase gene is present, it is indicated in the figure. The * refers to the full name of isolate L3_102_091G1: L3_102_091G1_dasL3_102_091G1_concoct_0. The figure was generated using iTOL and adapted with Adobe Illustrator 2024

The screening of a public garden opposite a companion animal hospital with a history of hospital-acquired infections and gut colonization with CPE, did not lead to the detection of any OXA-48-like producers as anticipated, but instead an *E. asburiae* producing the carbapenemase IMI-6 was isolated. In clinical settings, the IMI carbapenemases are still extremely rare in Switzerland [14]; they have already been associated with sporadic outbreaks in other countries and may increasingly disseminate due to their location on MGEs [1, 13, 15–16]. The strain, present near a trash bin in a public garden, was closely related to another *E. asburiae* isolated from a retail salad in the same country and to a strain from a rectal swab of a patient in France (Figs. 1 and 2), suggesting food as a possible dissemination vehicle. They all belonged to ST657, a lineage that has already been associated with clinical cases and carriage in humans across different countries, although it has not yet been frequently reported [13, 17]. The currently limited number of carbapenemase-producing *E. asburiae* reports and genome sequences does not allow the determination of the extend of the spread and their clinical significance. Nevertheless, the presence of closely related strains of a same lineage and harbouring carbapenemase-encoding plasmids in clinical and non-clinical settings suggests that exchange may occur between the different sectors, what may become a One Health concern. WGS-data are therefore essential to better characterize CPE and their MGEs in all sectors. Such data will provide baselines for epidemiological investigations and for tracing these so far neglected carbapenemase genes which have the potential to be acquired by various bacterial species, including those of clinical importance for both animals and humans.

## Electronic supplementary material

Below is the link to the electronic supplementary material.

Supplementary Material 1

## Data Availability

The complete hybrid assembly as well as the Illumina and Nanopore reads are deposited into GenBank under BioProject PRJNA1214994 with SRA acc. no. SRR32105527 and SRR32105526 and GenBank acc. no. CP179916 and CP179917.
