# Supplementary material for: Genomic comparative analysis of Enterobacter asburiae harbouring a conjugative blaIMI−6-plasmid isolated from a public garden in Switzerland
Source: Eur J Clin Microbiol Infect Dis. 2025 Apr 23;44(7):1739–44. doi: 10.1007/s10096-025-05131-7 (PMC12241205; doi:10.1007/s10096-025-05131-7)
Supplement: Supplementary file 1 — Supplementary Material 1 [file 10096_2025_5131_MOESM1_ESM.pdf]

European Journal of Clinical Microbiology & Infectious Diseases

Genomic characterization of *Enterobacter asburiae* harboring a conjugative bla_{IMI-6}-plasmid isolated from a public garden in Switzerland

Yvonne SPAHR[1], Andrea ENDIMIANI[2], Vincent PERRETEN[1*]

[1]Division of Molecular Bacterial Epidemiology & Infectious Diseases, Institute of Veterinary Bacteriology, Vetsuisse Faculty, University of Bern, Bern, Switzerland.

[2]Institute for Infectious Diseases (IFIK), University of Bern, Bern, Switzerland.

***Corresponding author**: Vincent Perreten, vincent.perreten@unibe.ch

Table S1: Sequencing statistics and genomic characteristics of *E. asburiae* strain 19YS-C

**Parameters**	**Results for 19YS-C**		
	**Total**	**Chromosome**	**p19YS-IMI-6**
**Origin**	Environment, Soil	-	-
**Region**	Bern, Switzerland	-	-
**GenBank acc. no.**[a]		CP179916	CP179917
**BioSample acc. no.**	SAMN46391438	-	-
**Illumina statistics**			
SRA acc. no.	SRR32105527	-	-
Read length	151	-	-
Number of paired reads	2,063,527	-	-
Coverage (x)	131	-	-
**Oxford Nanopore statistics**			
SRA acc. no.	SRR32105526	-	-
N50 (bp)	15,750	-	-
Mean read length (bp)	10,193.3	-	-
Number of reads	69,097	-	-
Coverage (x)	149	-	-
**Size and GC content**			
Total length (bp)	4,730,936	4,567,988	162,948
Coverage (x)	280	-	-
GC content (%)	55.99	56.1	52.8
**Number of:**			
Predicted genes	4,527	4,343	184
CDSs[b]	4,410	4,226	184
rRNAs[c]	25	25	0
tRNAs[d]	83	83	0
tmRNAs[e]	1	1	0
ARGs[f]	3	*bla*$_{ACT-10}$, *fos*A	*bla*$_{IMI-6}$

[a]acc. no. : Accession number

[b]CDS: Coding DNA sequences

[c]rRNA: ribosomal-RNA

[d]tRNA: transfer-RNA

[e]tmRNA: transfer-messenger-RNA

[f]ARG: Antimicrobial resistance gene
